# Supplementary material for: Efficient optical plasmonic tweezer-controlled single-molecule SERS characterization of pH-dependent amylin species in aqueous milieus
Source: Nat Commun. 2023 Nov 2;14:6996. doi: 10.1038/s41467-023-42812-3 (PMC10620188; doi:10.1038/s41467-023-42812-3)
Supplement: Supplementary file 1 — Supplementary Information [file 41467_2023_42812_MOESM1_ESM.pdf]

Supplementary information

Efficient optical plasmonic tweezer-controlled single-molecule SERS characterization of pH-dependent amylin species in aqueous milieus

Wenhao Fu†, Huanyu Chi†, Xin Dai, Hongni Zhu, Vince St. Dollente Mesias, Wei Liu*, and Jinqing Huang*

†These authors contributed equally to this work.

*Corresponding author. Email: wliu276@hku.hk (W. L.); jqhuang@ust.hk (J. H.)

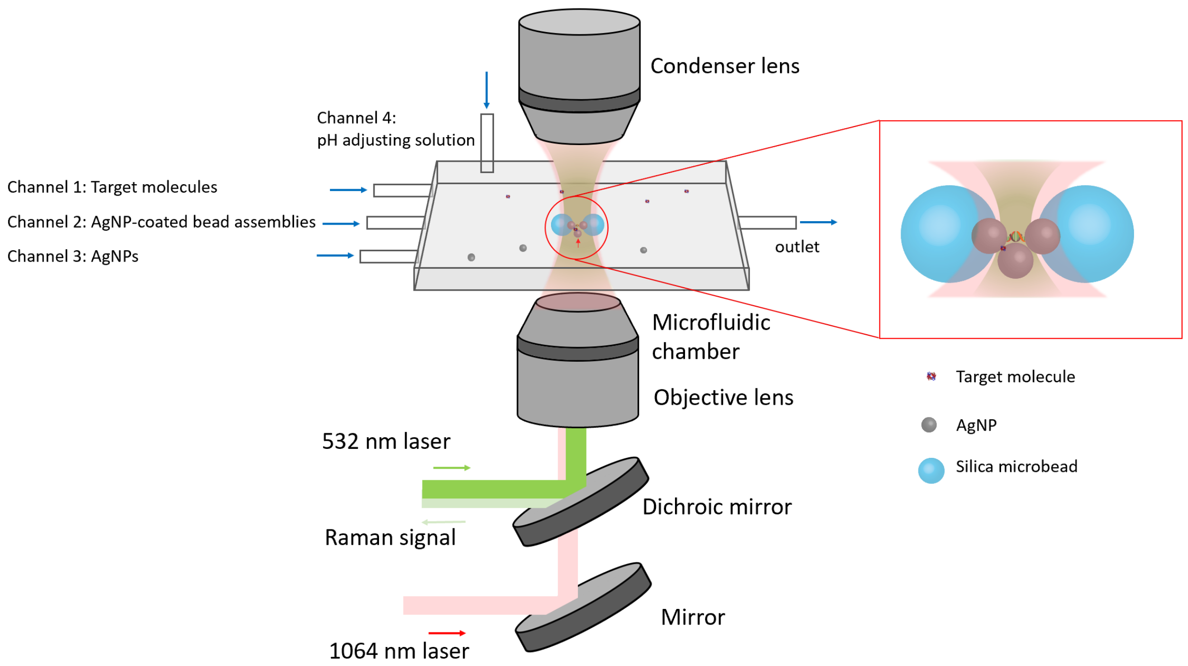

Supplementary Fig. 1. Instrumental setup of the optical plasmonic tweezers-coupled SERS platform.

a b

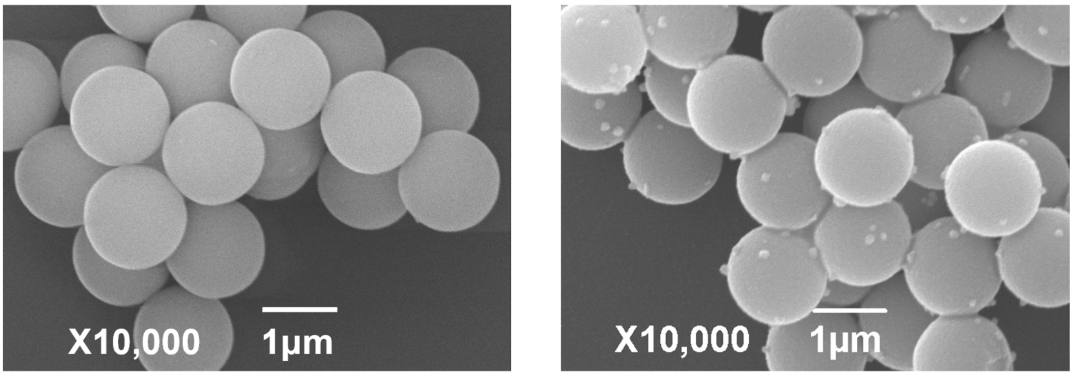

Supplementary Fig. 2. a Scanning electron microscope (SEM) image of silica microbeads. **b** SEM image of AgNP-coated silica microbeads. Representative images of three independent measurements are presented.

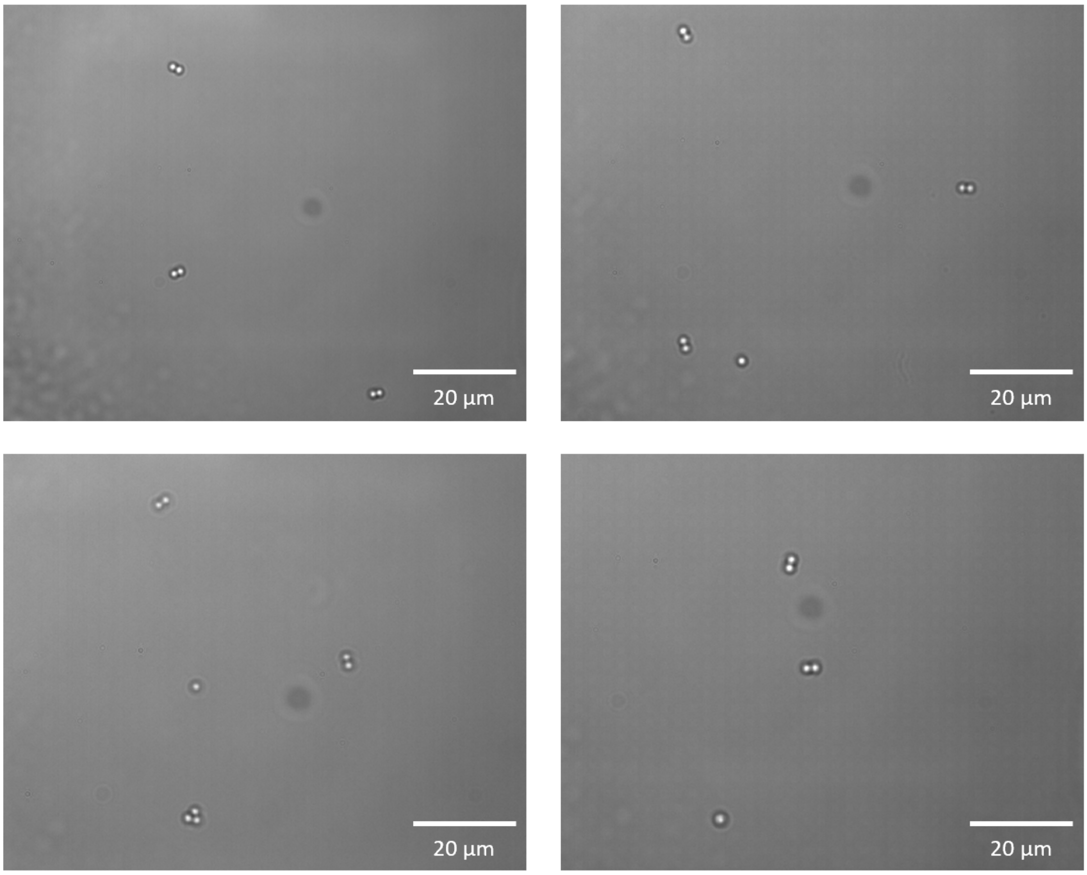

Supplementary Fig. 3. Brightfield images of the oligonucleotides-linked AgNP-coated bead assemblies deposited on the bottom surface of microfluidic chambers using the conventional microscope. Representative images of three independent measurements are presented.

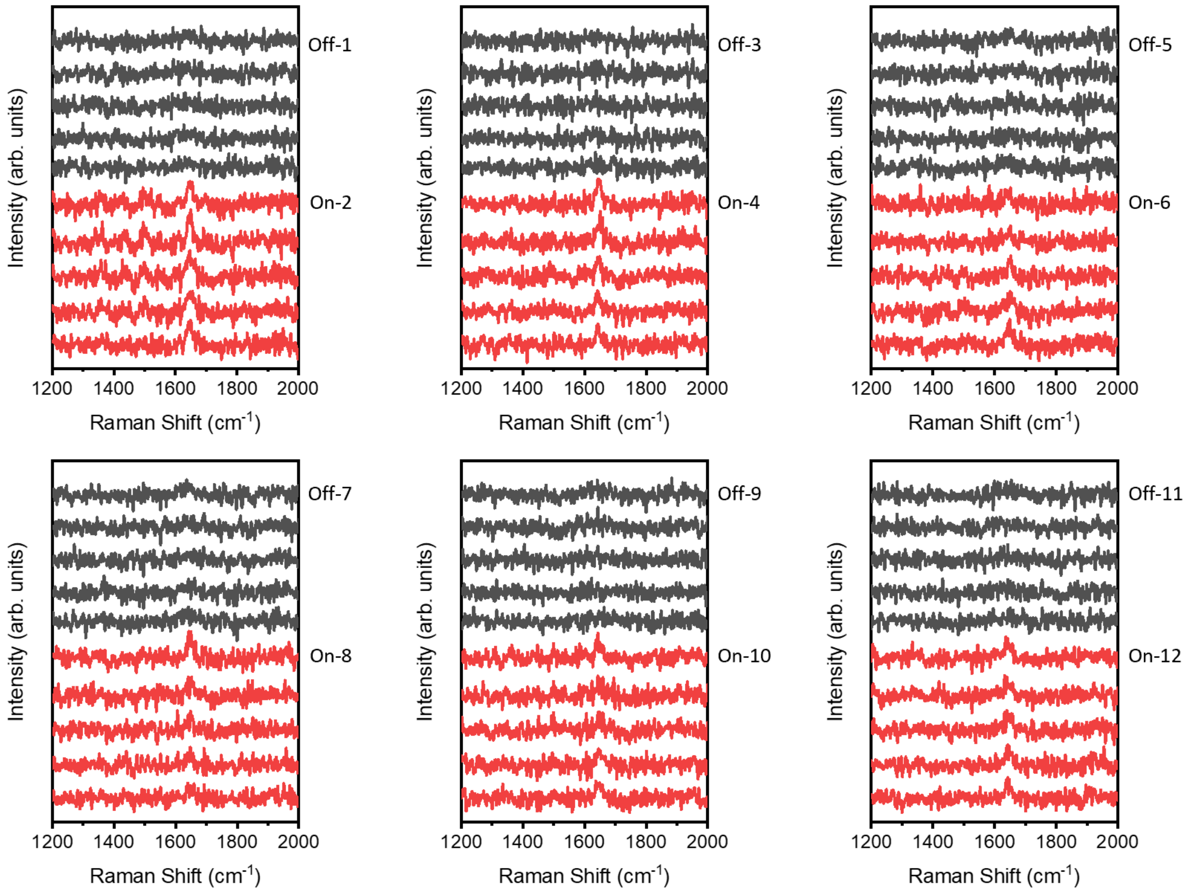

Supplementary Fig. 4. Real-time SERS spectra of 500 nM NBA acquired from the dynamic nanocavity during 12 times switching of the trapping laser. Black spectra were acquired at the off state and red spectra were acquired at the on state. Integration time: 1 s per spectrum. The term (arb. units) is abbreviated for arbitrary units.

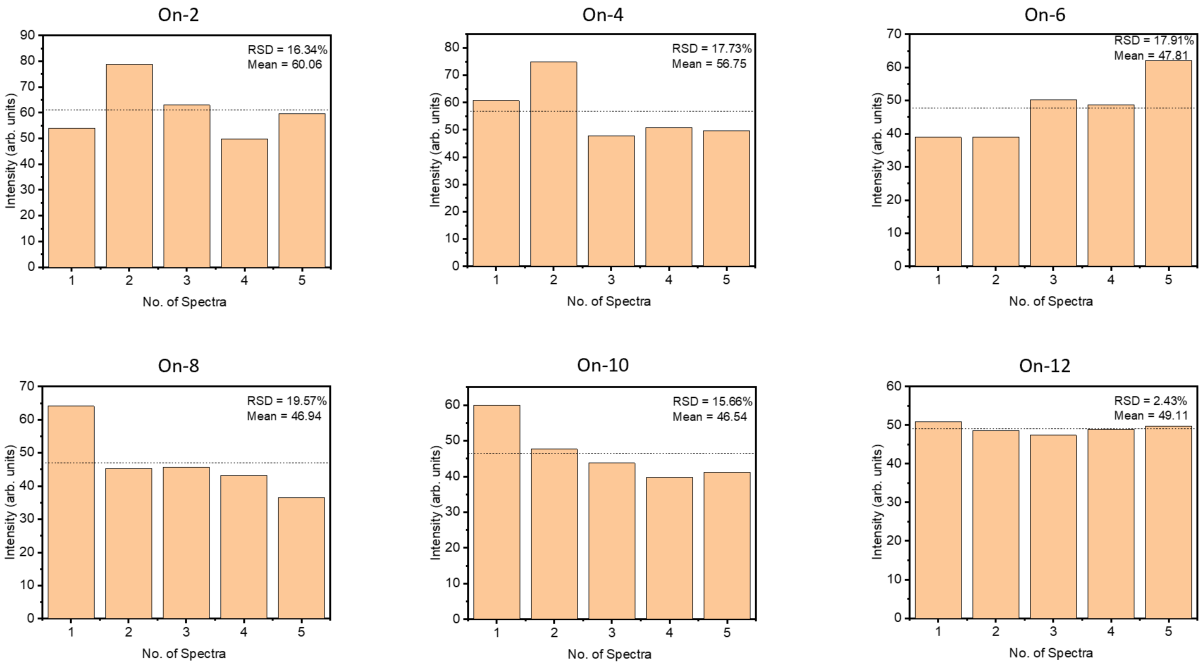

Supplementary Fig. 5. Histograms of peak intensities (1645 cm^{-1}) at each on state. Dot lines represent the average peak intensity at each on state. All the relative standard deviations (RSD) are within 20%, verifying the reproducibility of creating the dynamic nanocavity on our platform. The term (arb. units) is abbreviated for arbitrary units.

Supplementary Note 1. 3D-FDTD Simulation

Three-dimensional finite-difference time-domain (3D-FDTD) simulation was performed using Lumerical FDTD SOLUTIONS to simulate the intensity and distribution of electromagnetic field and caluculate the trapping force and trapping potential. A simplified model was constructed consisting of a pair of dimeric AgNP (70 nm) coated on the junction between two silica beads (1.26 μm). The interparticle gap was set as 10 nm. The dielectric properties of Ag and SiO_2 were taken from Johnson & Christy database and Palik database, respectively. The refractive index of background fluid was set as 1.33. A 532 nm Gaussian wave and a 1064 nm Gaussian wave with polarization parallel to the AgNP-coated beads dimer was injected along the z-axis. Perfectly matched layer (PML) boundary condition was used, and the mesh size in the interparticle gap was set as 0.2 nm to increase the accuracy of the simulation. An overall mesh setting with an accuracy of 5 was applied for the rest region.

Trapping force simulation

The force exerted on a AgNP in a harmonic electromagnetic field can be derived using the Maxwell Stress Tensor (MST)[1–3]. By integrating the MST over a closed cubic surface surrounding the particle, the time-averaged force can be calculated by:

$$\mathbf{F} = \int_V \mathbf{f}\, d\tau = -\oint_S \overleftrightarrow{\mathbf{T}} \cdot \mathbf{n}\, dS$$

Where $\mathbf{F}$ is the time averaged optical force, V is the volume of AgNP, $\mathbf{f}$ is the force density, τ is the unit volume. S is the surface surrounding the particle, $\mathbf{n}$ is the unit normal vector to the surface, and $\overleftrightarrow{\mathbf{T}}$ is the Maxwell Stress Tensor which can be expressed as:

$$\overleftrightarrow{\mathbf{T}} = \varepsilon_0 \varepsilon \mathbf{EE} + \mu_0 \mu \mathbf{HH} - \frac{1}{2}(\varepsilon_0 \varepsilon E^2 + \mu_0 \mu H^2)\overleftrightarrow{\mathbf{I}}$$

where $\mathbf{EE}$ and $\mathbf{HH}$ represent the outer products of electric field and magnetic field, $\overleftrightarrow{\boldsymbol{I}}$ denotes the unit tensor, and ε and μ are the dielectric permittivity and magnetic permeability of the surrounding medium, respectively.

Since the diameter of AgNP is much smaller than the wavelength of light, the AgNP itself can be treated as a point dipole with polarizability $\alpha = \alpha' + i\alpha'' = 3V\frac{\varepsilon_p - \varepsilon_m}{\varepsilon_p + 2\varepsilon_m}$. In this case, the trapping potential can be calculated by:

$$U = -\int \mathbf{F} dr = -\frac{\alpha'}{2}|\mathbf{E}|^2$$

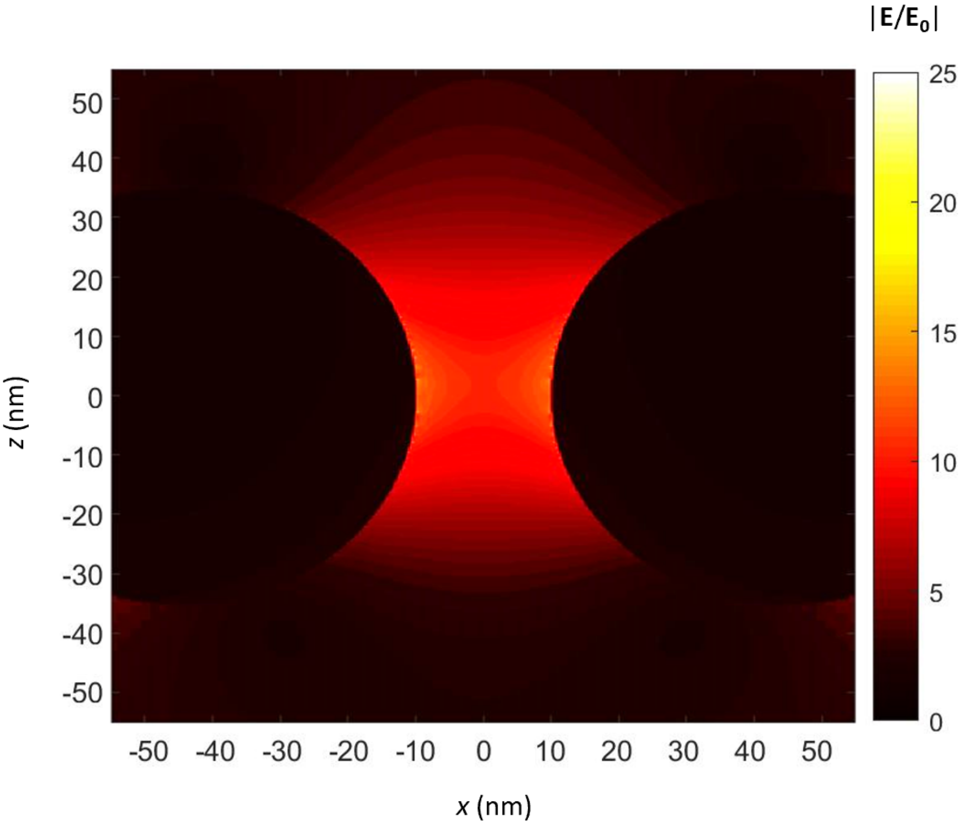

Supplementary Fig. 6. FDTD simulation of the electric field distribution in the vicinity of the AgNP-coated bead dimer upon excitation of plasmonic resonance. The region with most intense field intensity locates in the junction between two beads.

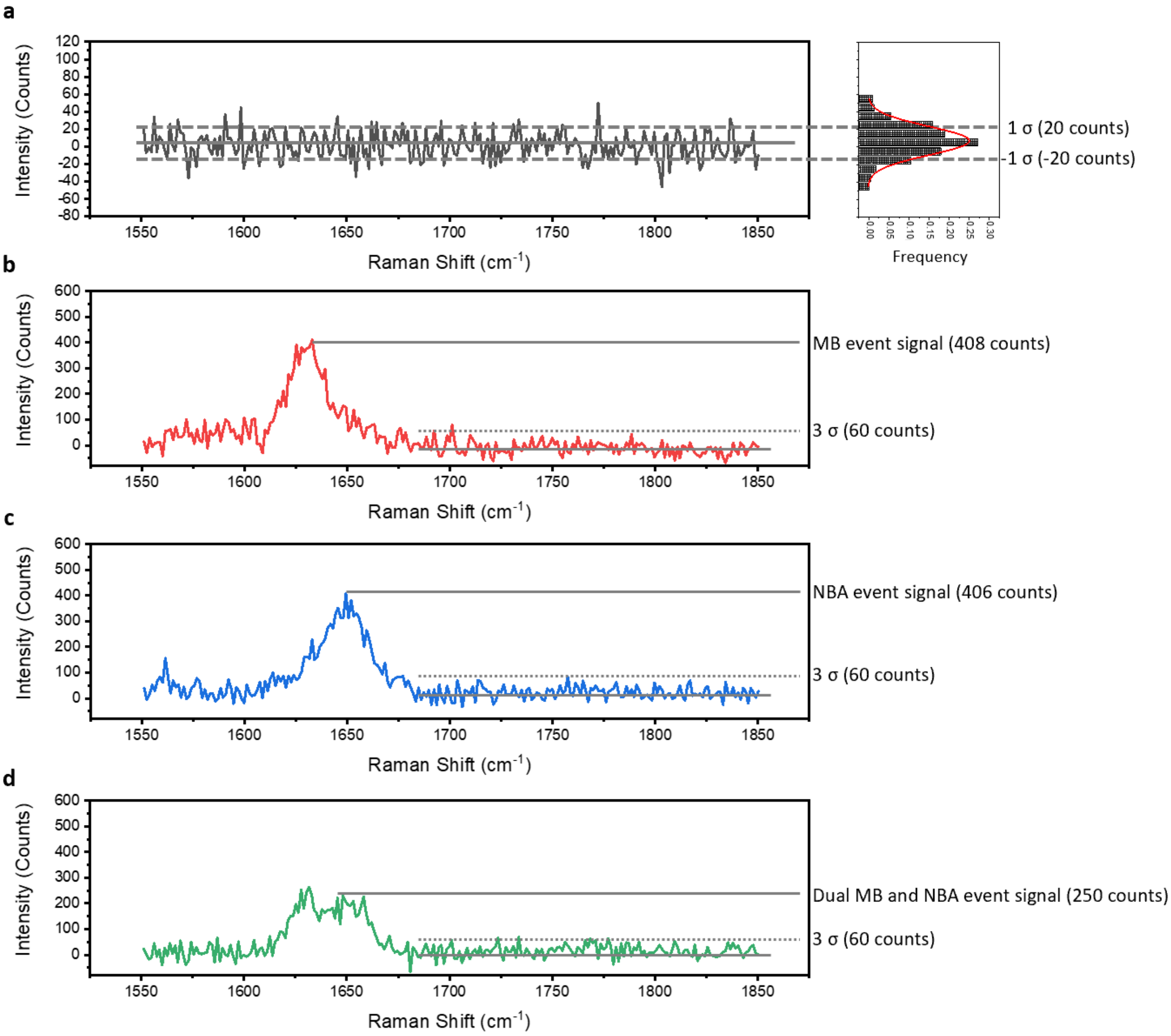

Supplementary Fig. 7. **a** Original background spectrum and noise analysis, giving the standard deviation 1 σ as 20 counts (-1 σ as -20 counts). **b** Representative spectrum of single-MB event. **c** Representative spectrum of single-NBA event. **d** Representative spectrum of dual-MB and NBA event, showing the intensity cutoff criterion for distinguishing signals from background noise at 3 σ as 60 counts.

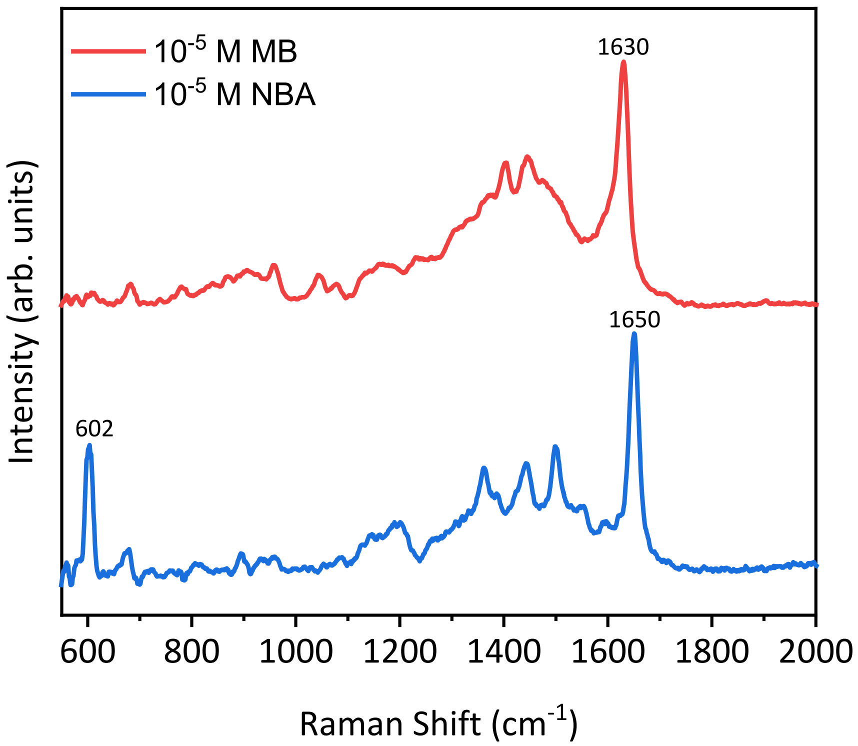

Supplementary Fig. 8. SERS spectra of MB (red) and NBA (blue) at the concentration of 10^{-5} M as the reference. The Raman characteristic peaks for the identification of MB and NBA events are marked. The term (arb. units) is abbreviated for arbitrary units.

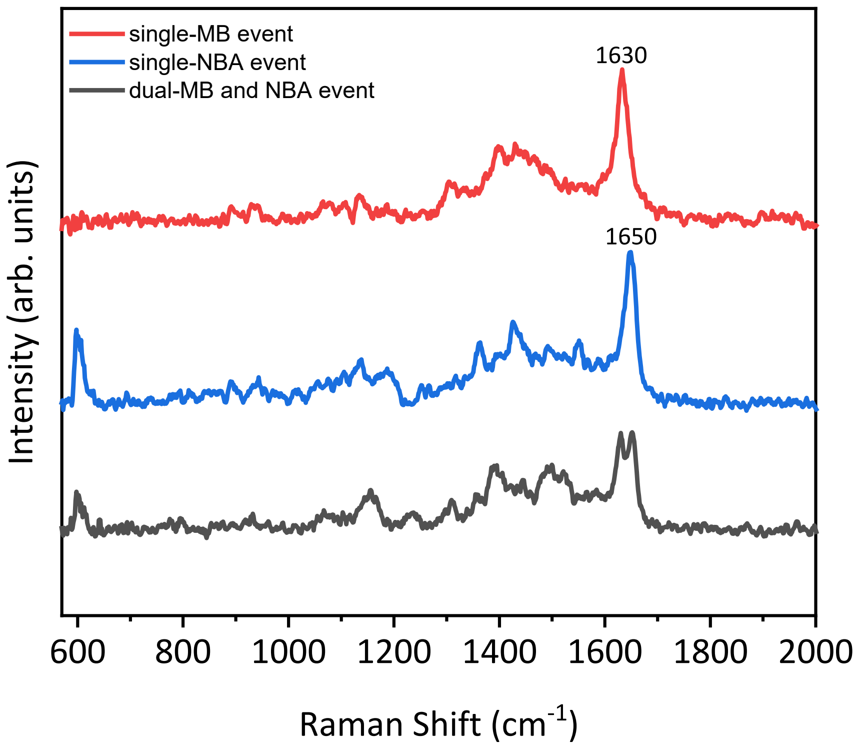

Supplementary Fig. 9. Representative full-ranged spectra of single-MB event (red), single-NBA event (blue) and dual-MB and NBA event (black), corresponding to the spectra shown in Fig. 2b. The term (arb. units) is abbreviated for arbitrary units.

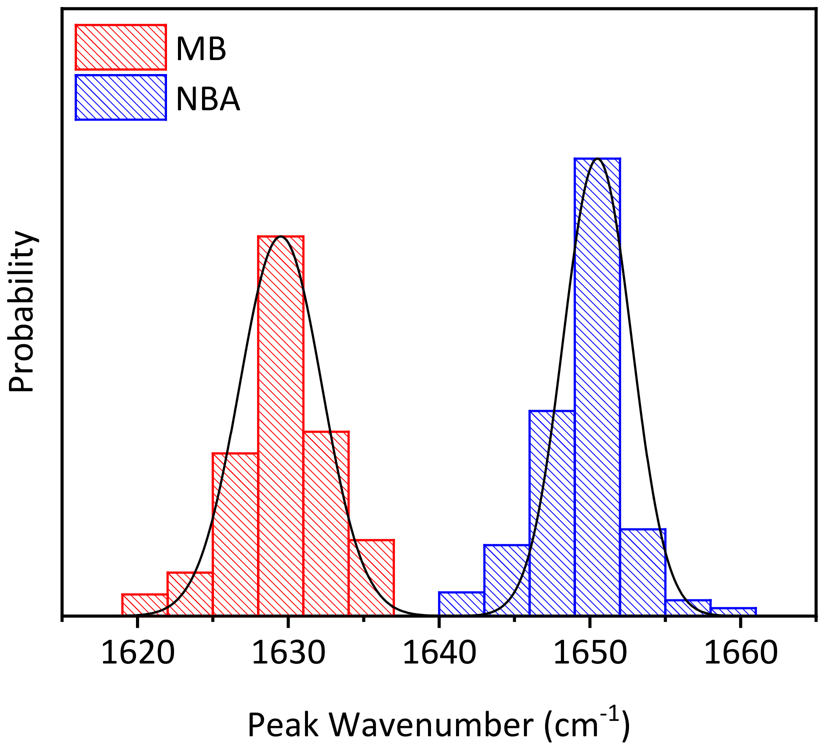

Supplementary Fig. 10. Distribution of the characteristic peak positions of single-MB events (red), and single-NBA events (blue) in the BiASERS experiment.

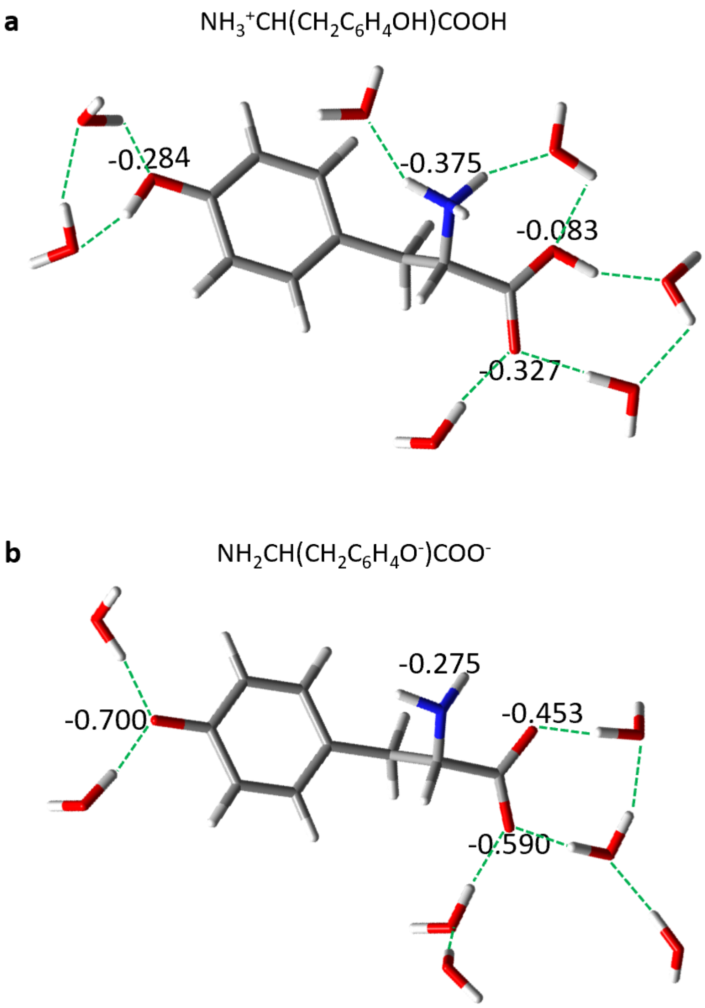

Supplementary Fig. 11. a Sketch map of Tyrosine (+1) + 7 H_2O cluster. **b** Sketch map of Tyrosinate (-2) + 7 H_2O cluster. Dotted green lines represent hydrogen-bonds. The numbers denote the calculated charge of nitrogen/oxygen.

Supplementary Note 2. Density functional theory (DFT) calculation

DFT calculations were performed using the Gaussian 09 software package. The geometry optimizations and vibrational frequencies of the Tyr-H_2O clusters were computed using the hybrid B3LYP density functional level along with 6-311++ G(d,p) basis set. The construction of Tyr-H_2O clusters was initially based on B. Hernández's work[4] by involving seven water molecules in the vicinity to simulate the hydration states of the amino group, carboxyl group, and phenol hydroxyl group of Tyr. Electrostatic potential (ESP) analysis of Tyr (+1) and Tyr (-2) molecules was carried out by Multiwfn 3.7 code[5] and depicted by the VDM visualization program[6], respectively.

Supplementary Note 3. Least-squares procedures for wavenumber corrections

Based on a least-squares procedure[7,8], the optimum scaling factor λ that correlates calculated frequencies to experimental wavenumbers is determined by minimizing the residual Δ:

$$\Delta = \sum_{i}^{N} \left(\lambda \omega_i^{cal} - \widetilde{\omega}_i^{exp}\right)^2$$

Where λ is the scaling factor. N is the total number of vibrational modes in a designated wavenumber region. ω_i^{cal} and $\widetilde{\omega}_i^{exp}$ are the i th calculated frequencies and experimental wavenumbers, respectively.

By substituting the calculated frequencies and the experimental wavenumbers of Tyr at +1 charged state under pH 1.0 condition in the high wavenumber region (1000-2000 cm^{-1}) into the equation, we can get:

$$\begin{aligned}\Delta_1 = {} & (1674\lambda_1 - 1620)^2 + (1604\lambda_1 - 1572)^2 + (1508\lambda_1 - 1434)^2 + (1380\lambda_1 - 1352)^2 \\ & + (1350\lambda_1 - 1333)^2 + (1308\lambda_1 - 1271)^2 + (1236\lambda_1 - 1217)^2 \\ & + (1150\lambda_1 - 1153)^2 + (1082\lambda_1 - 1073)^2\end{aligned}$$

This function is quadratic in nature and its graphical representation forms a parabolic curve, as shown in Supplementary Fig. 12:

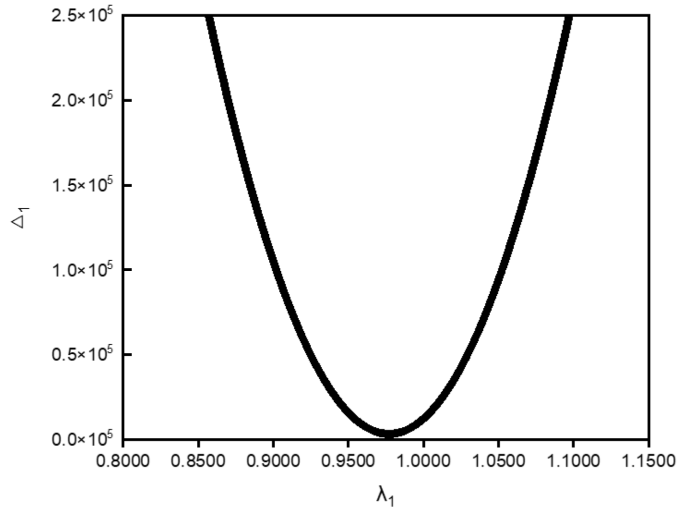

Supplementary Fig. 12. Residues as a function of scaling factors correlating the calculated frequencies and the experimental wavenumbers of Tyr at +1 charged state under pH 1.0 condition in the high wavenumber region (1000-2000 cm^{-1}).

Δ_1 reaches its minimum at the vertex of the quadratic function, denoted as $\frac{d\Delta_1}{d\lambda_1} = 0$, leading to:

$$\lambda_{1\,opt} = \frac{\sum_i^N(\omega_i^{cal}\tilde{\omega}_i^{exp})}{\sum_i^N(\omega_i^{cal})^2}$$

$$= \frac{1674 \times 1620 + 1604 \times 1572 + 1508 \times 1434 + 1380 \times 1352 + 1350 \times 1333 + 1308 \times 1271 + 1236 \times 1217 + 1150 \times 1153 + 1082 \times 1073}{1674^2 + 1604^2 + 1508^2 + 1380^2 + 1350^2 + 1308^2 + 1236^2 + 1150^2 + 1082^2}$$

$$= 0.9770$$

Therefore, the optimum scaling factor $\lambda_{1\,opt}$ that correlates the calculated frequencies and the experimental wavenumbers of Tyr at +1 charged state under pH 1.0 condition is 0.9770.

Similarly, by substituting the calculated frequencies and the experimental wavenumbers of Tyr at -2 charged state under pH 13.0 condition in the high wavenumber region (1000-2000 cm^{-1}) into the equation, we can get:

$$\Delta_2 = (1630\lambda_2 - 1602)^2 + (1594\lambda_2 - 1570)^2 + (1526\lambda_2 - 1442)^2 + (1480\lambda_2 - 1423)^2 + (1352\lambda_2 - 1338)^2 + (1290\lambda_2 - 1269)^2 + (1222\lambda_2 - 1216)^2 + (1182\lambda_2 - 1161)^2 + (1092\lambda_2 - 1071)^2$$

This quadratic function gives a parabolic graph as shown in Supplementary Fig. 13:

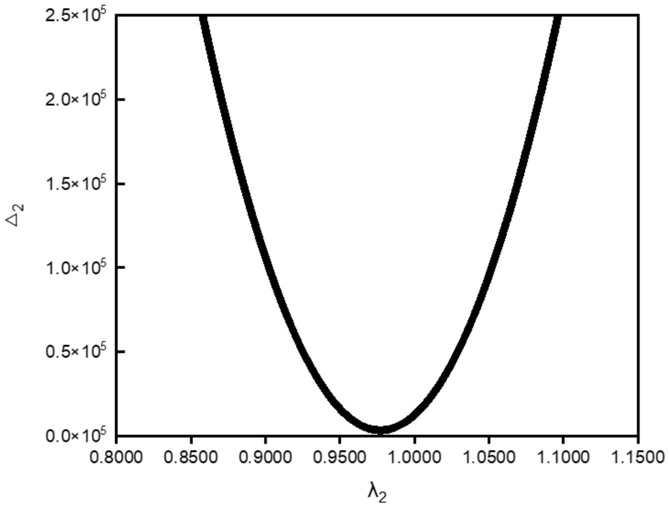

Supplementary Fig. 13. Residues as a function of scaling factors correlating the calculated frequencies and the experimental wavenumbers of Tyr at -2 charged state under pH 13.0 condition in the high wavenumber region (1000-2000 cm^{-1}).

Δ_2 reaches its minimum at the vertex of the quadratic function, denoted as $\frac{d\Delta_2}{d\lambda_2} = 0$, leading to:

$$\lambda_{2\ opt} = \frac{\sum_i^N (\omega_i^{cal} \widetilde{\omega}_i^{exp})}{\sum_i^N (\omega_i^{cal})^2}$$

$$= \frac{\begin{matrix} 1630 \times 1602 + 1594 \times 1570 + 1526 \times 1442 + 1480 \times 1423 + \\ 1352 \times 1338 + 1290 \times 1269 + 1222 \times 1216 + 1182 \times 1161 + 1092 \times 1071 \end{matrix}}{1630^2 + 1594^2 + 1526^2 + 1480^2 + 1352^2 + 1290^2 + 1222^2 + 1182^2 + 1092^2}$$

$$= 0.9770$$

Therefore, the optimum scaling factor $\lambda_{2\ opt}$ that correlates the calculated frequencies and the experimental wavenumbers of Tyr at -2 charged state under pH 13.0 condition is 0.9770.

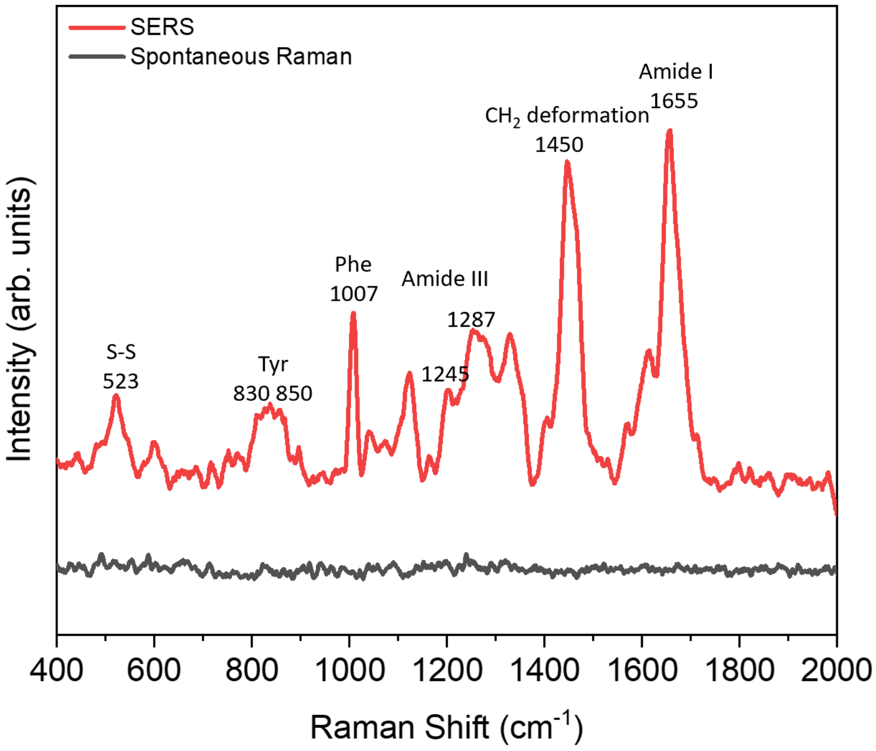

Supplementary Fig. 14. Spontaneous Raman spectrum (black) and SERS spectrum (red) of 10 μM hIAPP, showing the SERS enhancement to overcome the detection threshold of spontaneous Raman spectroscopy. The SERS spectrum (red) of 10 μM hIAPP is the same as the data shown in Fig. 4c. The term (arb. units) is abbreviated for arbitrary units.

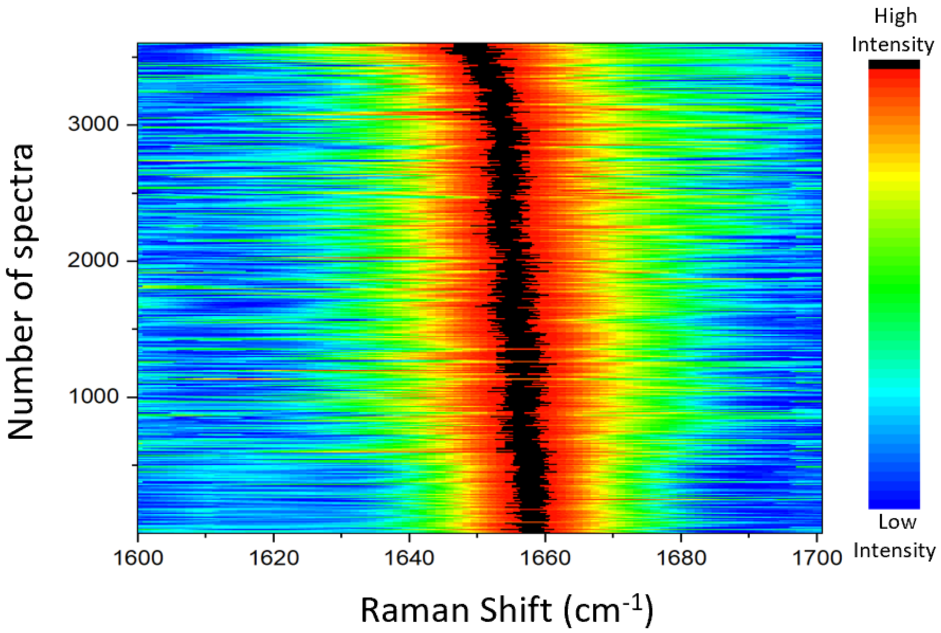

Supplementary Fig. 15. Mapping of secondary derivative spectra of the amide I region of the SERS spectra of hIAPP at $t = 0$ h incubation under pH 5.5 from parallel experimental sessions. The color bar shows the normalized intensities from low (dark blue) to high (red-black).

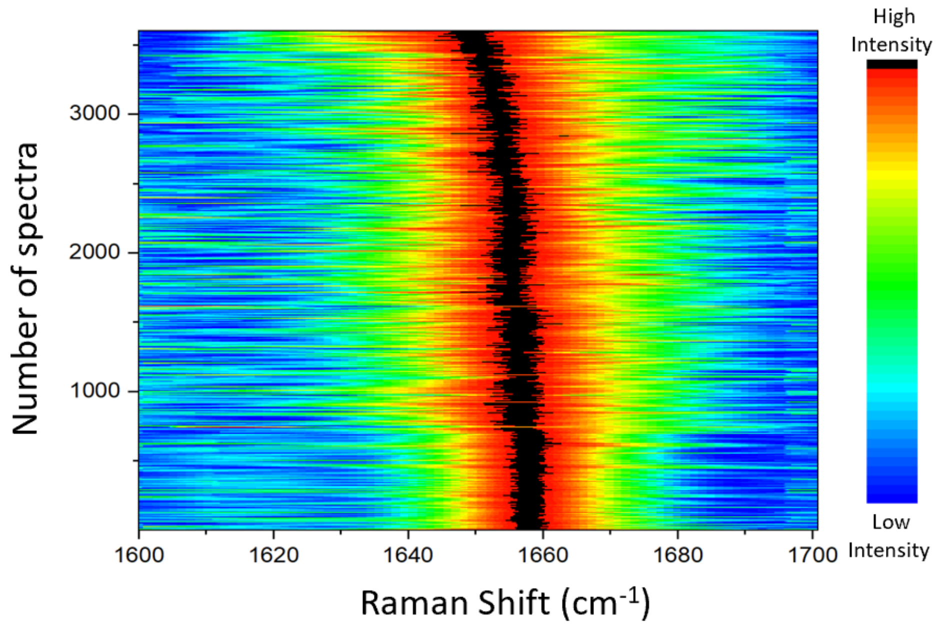

Supplementary Fig. 16. Mapping of secondary derivative spectra of the amide I region of the SERS spectra of hIAPP at t = 0 h incubation under pH 7.4 from parallel experimental sessions. The color bar shows the normalized intensities from low (dark blue) to high (red-black).

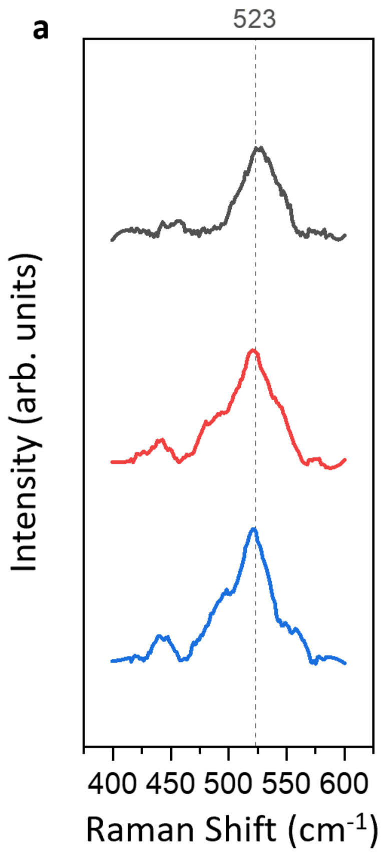

Supplementary Fig. 17. a Representative SERS spectra of hIAPP before the incubation at the concentration of 10 µM under pH 7.4 condition. **b** Representative SERS spectra of hIAPP after the incubation at the concentration of 10 µM under pH 7.4 condition for 24 hours, showing the disulfide bond between Cys2-Cys7 of hIAPP in the region of 400-600 cm^{-1}. The term (arb. units) is abbreviated for arbitrary units.

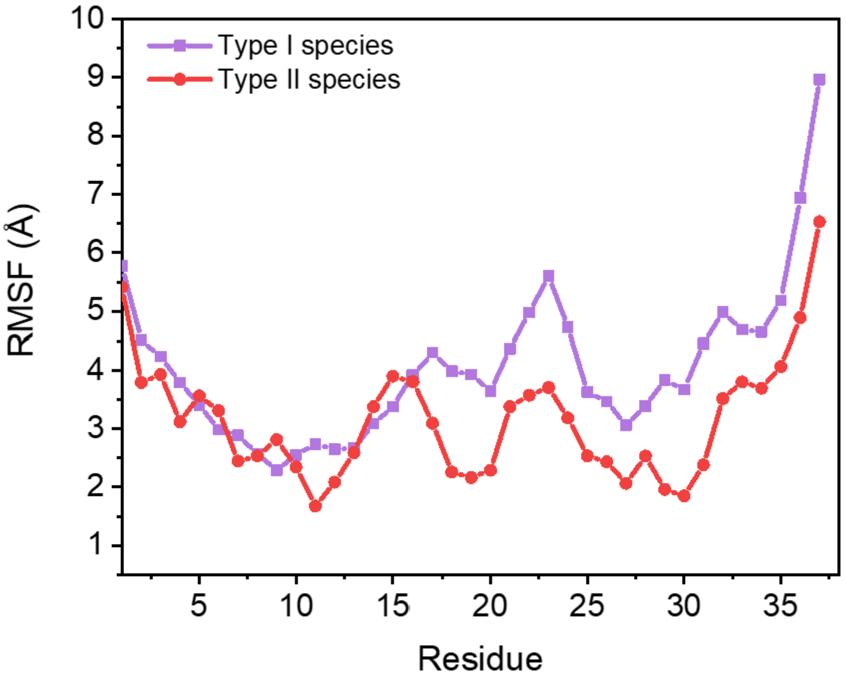

Supplementary Fig. 18. Root-mean-square fluctuation (RMSF) of the type I (purple) and type II (red) transient species. The RMSF is evaluated over the protein backbone atoms. For the C-terminal residues (30-37), the RMSF values of type II species are lower than those of type I species, suggesting that the type II species adopt a more constrained C-terminal.

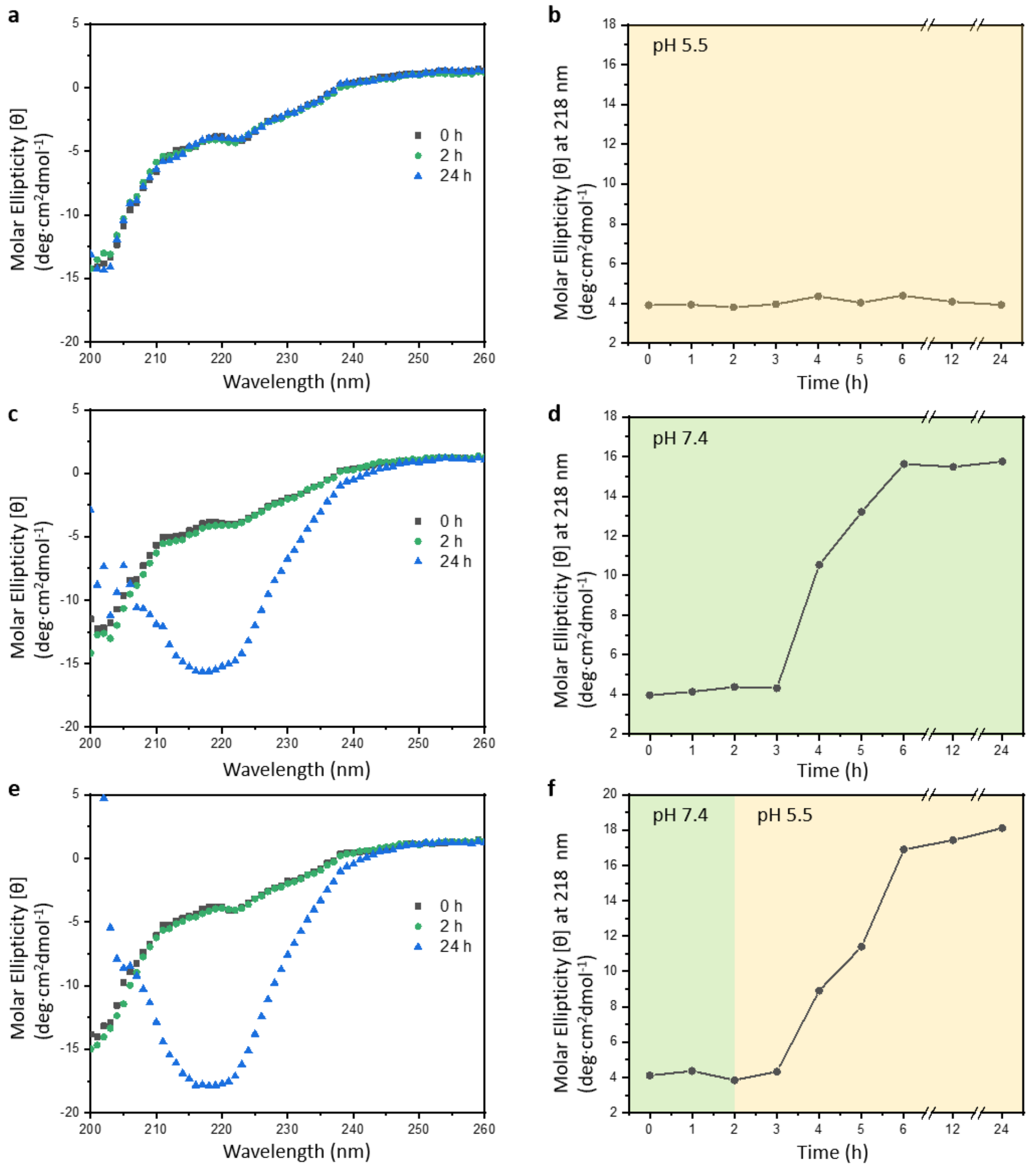

Supplementary Fig. 19. a CD spectra of 10 μM hIAPP solution under pH 5.5 incubation at t = 0, 2, and 24 h. **b** The absolute intensities at 218 nm of the CD spectra as a function of time during the incubation of hIAPP under pH 5.5 for 24 hours. **c** CD spectra of 10 μM hIAPP solution under pH 7.4 incubation at t = 0, 2, and 24 h. **d** The absolute intensities at 218 nm of the CD spectra as a function of time during the incubation of hIAPP under pH 7.4 for 24 hours. **e** CD spectra of 10 μM hIAPP solution at t = 0, 2, and 24 h in the incubation under pH 7.4 for the first 2 hours and then adjusted to pH 5.5 for the following 22 hours. **f** The absolute intensities at 218 nm of the CD spectra as a function of time during the incubation of hIAPP under pH 7.4 for the first 2 hours, followed by the adjustment from pH 7.4 to 5.5 to continue the incubation for the succeeding 22 hours.

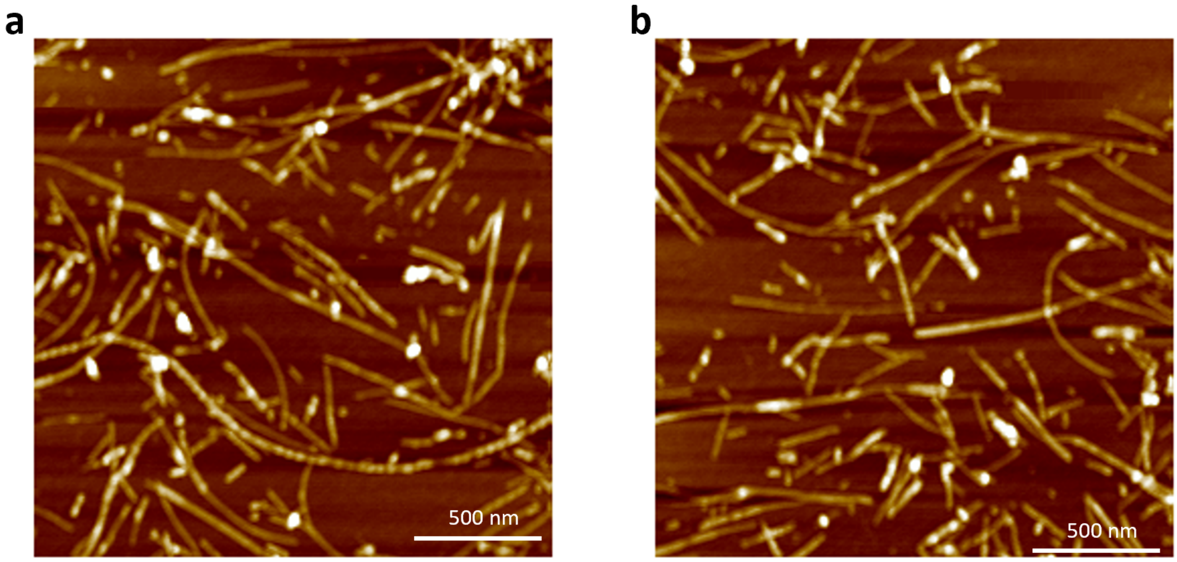

Supplementary Fig. 20. a AFM image of the amyloid fibrils generated after the 24-hour incubation of hIAPP at pH 7.4. **b** AFM image of the amyloid fibrils generated after the 24-hour incubation of hIAPP with the initial incubation at pH 7.4 for 2 hours, followed by the adjustment of pH from 7.4 to 5.5 to continue the subsequent incubation at pH 5.5 for the succeeding 22 hours. Representative images of three independent measurements are presented.

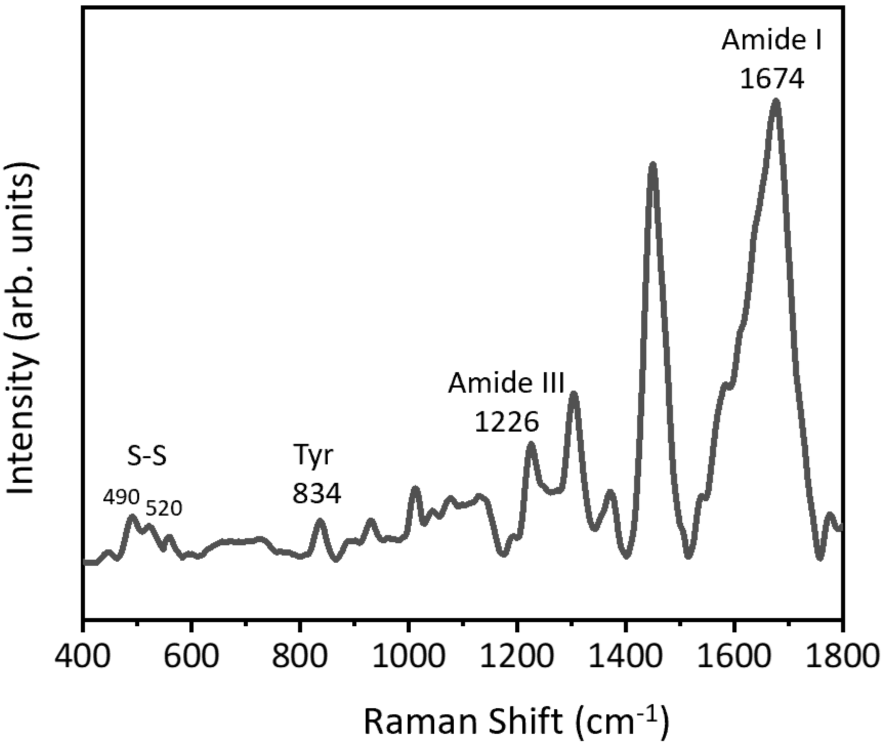

Supplementary Fig. 21. SERS spectrum of the amyloid fibrils generated after the 24-hour incubation of hIAPP with the initial incubation at pH 7.4 for 2 hours, followed by the adjustment of pH from 7.4 to 5.5 to continue the subsequent incubation at pH 5.5 for the succeeding 22 hours. The term (arb. units) is abbreviated for arbitrary units.

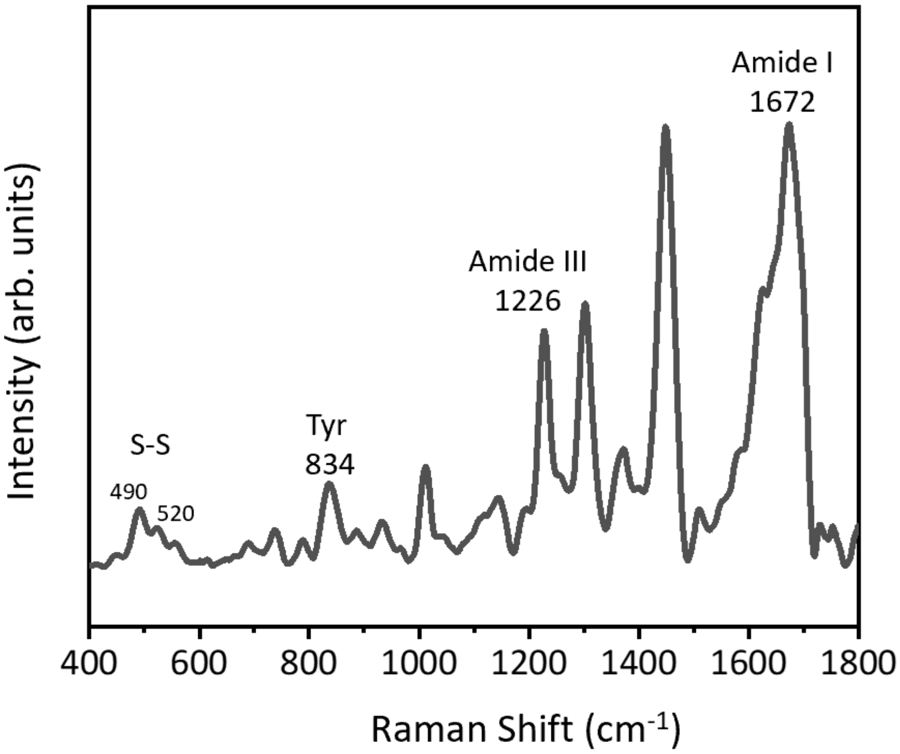

Supplementary Fig. 22. SERS spectrum of the amyloid fibrils generated after the 24-hour incubation of hIAPP at pH 7.4. The term (arb. units) is abbreviated for arbitrary units.

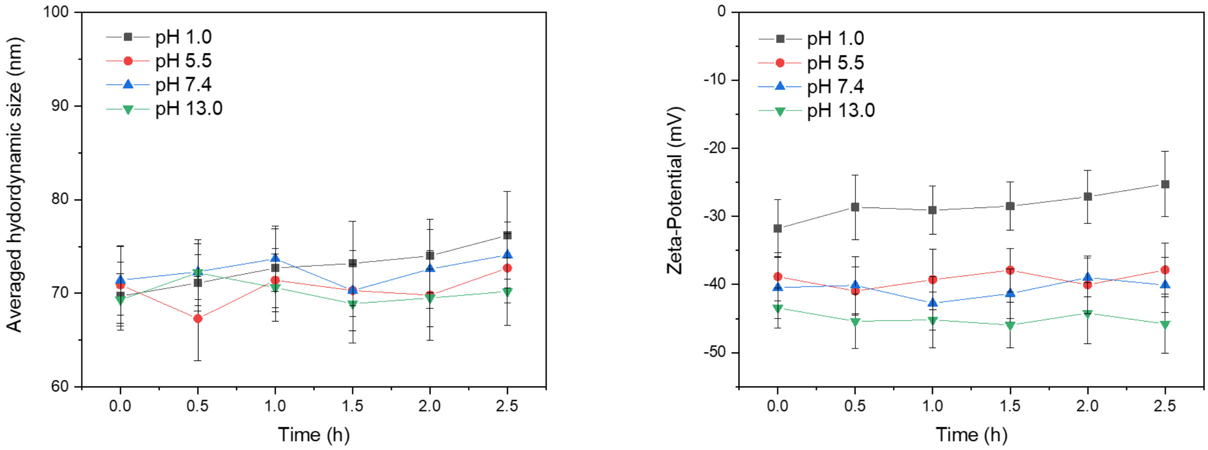

Supplementary Fig. 23. Plots of the time evolution of the average hydrodynamic diameters and the average zeta-potential of 10^{-11} M AgNP under different pH conditions. Data are shown as mean with errors derived from three parallel measurements.

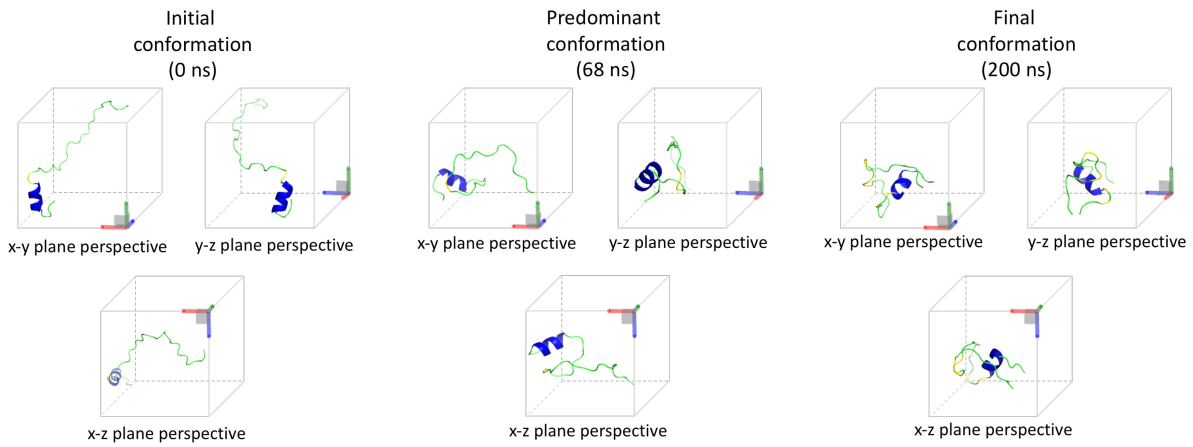

Supplementary Fig. 24. Snapshots from *x-y*, *y-z*, and *x-z* plane perspectives of the initial conformation, the predominant conformation and the final conformation in the representative simulation trajectory of monomeric hIAPP with +4 charges at pH 5.5 shown in Fig. 4j.

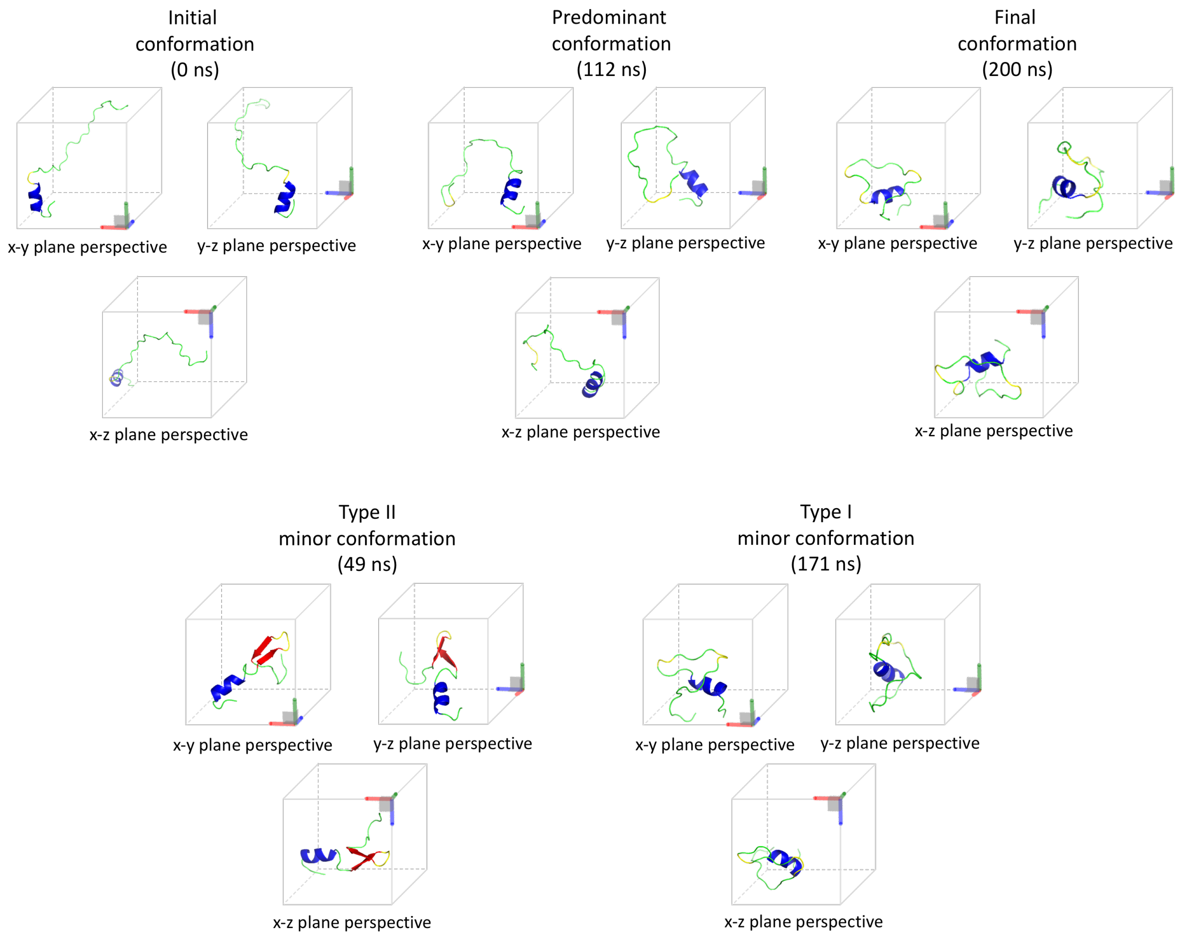

Supplementary Fig. 25. Snapshots from *x-y*, *y-z*, and *x-z* plane perspectives of the initial conformation, the type II minor conformation, the predominant conformation, the type I minor conformation, and the final conformation in the representative simulation trajectory of monomeric hIAPP with +3 charges at pH 7.4 shown in Fig. 5j.

Supplementary Table 1. The vibrational bands assignment of hIAPP.

Raman Shift (cm^{-1})	Assignment*
836, 854	Tyr doublet
899	*v*(C-C)
946	*v*(C-C)
1010	Phe
1090	*v*(C-C), *v*(C-N), *v*(C-O)
1135	*v*(C-C)
1211	Tyr
1220-1240, β-sheet	
1240-1250, Random coil	Amide III
1270-1300, α-helix	
1310	CH_2 deformation
1366	CH_2 deformation
1450	CH_2, CH_3 deformation
1604	Phe
1612	Tyr
1650-1660, helix-coil	
1660-1670, turn	Amide I
1670-1680, β-sheet	

*Assignments are based on References[9–11].

Supplementary References

1. Kotsifaki, D. G., Truong, V. G. & Nic Chormaic, S. Plasmon-Enhanced Optical Forces and Tweezers. in *Plasmon-enhanced light-matter interactions* (eds. Yu, P., Xu, H. & Wang, Z. M.) 177–206 (Springer International Publishing, 2022). doi:10.1007/978-3-030-87544-2_8.
2. Min, C. *et al.* Focused plasmonic trapping of metallic particles. *Nat. Commun.* **4**, 1–7 (2013).
3. Hong, S., Shim, O., Kwon, H. & Choi, Y. Autoenhanced Raman Spectroscopy via Plasmonic Trapping for Molecular Sensing. *Anal. Chem.* **88**, 7633–7638 (2016).
4. Hernández, B., Coïc, Y. M., Pflüger, F., Kruglik, S. G. & Ghomi, M. All characteristic Raman markers of tyrosine and tyrosinate originate from phenol ring fundamental vibrations. *J. Raman Spectrosc.* **47**, 210–220 (2016).
5. Lu, T. & Chen, F. Multiwfn: A multifunctional wavefunction analyzer. *J. Comput. Chem.* **33**, 580–592 (2012).
6. Humphrey, W., Dalke, A. & Schulten, K. VMD: Visual Molecular Dynamics. *J. Mol. Graph.* **14**, 33–38 (1996).
7. Scott, A. P. & Radom, L. Harmonic Vibrational Frequencies: An Evaluation of Hartree−Fock, Møller−Plesset, Quadratic Configuration Interaction, Density Functional Theory, and Semiempirical Scale Factors. *J. Phys. Chem.* **100**, 16502–16513 (1996).
8. Palafox, M. A. DFT computations on vibrational spectra: Scaling procedures to improve the wavenumbers. *Phys. Sci. Rev.* **3**, 1–30 (2019).
9. Rygula, A. *et al.* Raman spectroscopy of proteins: A review. *J. Raman Spectrosc.* **44**, 1061–1076 (2013).
10. Louros, N. N. *et al.* Tracking the amyloidogenic core of IAPP amyloid fibrils: Insights from micro-Raman spectroscopy. *J. Struct. Biol.* **199**, 140–152 (2017).
11. Sadat, A. & Joye, I. J. Peak fitting applied to fourier transform infrared and raman spectroscopic analysis of proteins. *Appl. Sci.* **10**, (2020).
