# Supplementary material for: Efficient optical plasmonic tweezer-controlled single-molecule SERS characterization of pH-dependent amylin species in aqueous milieus
Source: Nat Commun. 2023 Nov 2;14:6996. doi: 10.1038/s41467-023-42812-3 (PMC10620188; doi:10.1038/s41467-023-42812-3)
Supplement: Supplementary file 2 — Description of Additional Supplementary Files [file 41467_2023_42812_MOESM2_ESM.pdf]

Description of Additional Supplementary Files:

Supplementary Data 1: PDB file encompassing all conformations extracted from the representative simulation trajectory of monomeric hIAPP with +4 charges at pH 5.5 shown in Fig. 4i.

Supplementary Data 2: PDB file encompassing all conformations extracted from the representative simulation trajectory of monomeric hIAPP with +3 charges at pH 7.4 shown in Fig. 5i.

Supplementary Movie 1: Video demonstrating the structural dynamics of monomeric hIAPP with +4 charges at pH 5.5 in the representative simulation trajectory shown in Fig. 4i.

Supplementary Movie 2: Video demonstrating the 360-degree rotations of the initial conformation (0 ns) of monomeric hIAPP with +4 charges at pH 5.5 shown in Fig. 4j.

Supplementary Movie 3: Video demonstrating the 360-degree rotations of the predominant conformation (68 ns) of monomeric hIAPP with +4 charges at pH 5.5 shown in Fig. 4j.

Supplementary Movie 4: Video demonstrating the 360-degree rotations of the final confirmation (200 ns) of monomeric hIAPP with +4 charges at pH 5.5 shown in Fig. 4j.

Supplementary Movie 5: Video demonstrating the structural dynamics of monomeric hIAPP with +3 charges at pH 7.4 in the representative simulation trajectory shown in Fig. 5i.

Supplementary Movie 6: Video demonstrating the 360-degree rotations of the initial conformation (0 ns) of monomeric hIAPP with +3 charges at pH 7.4 shown in Fig. 5j.

Supplementary Movie 7: Video demonstrating the 360-degree rotations of the type II minor conformation (49 ns) of monomeric hIAPP with +3 charges at pH 7.4 shown in Fig. 5j.

Supplementary Movie 8: Video demonstrating the 360-degree rotations of the predominant conformation (112 ns) of monomeric hIAPP with +3 charges at pH 7.4 shown in Fig. 5j.

Supplementary Movie 9: Video demonstrating the 360-degree rotations of the type I minor conformation (171 ns) of monomeric hIAPP with +3 charges at pH 7.4 shown in Fig. 5j.

Supplementary Movie 10: Video demonstrating the 360-degree rotations of the final confirmation (200 ns) of monomeric hIAPP with +3 charges at pH 7.4 shown in Fig. 5j.
