# Supplementary material for: Efficient optical plasmonic tweezer-controlled single-molecule SERS characterization of pH-dependent amylin species in aqueous milieus
Source: Nat Commun. 2023 Nov 2;14:6996. doi: 10.1038/s41467-023-42812-3 (PMC10620188; doi:10.1038/s41467-023-42812-3)
Supplement: Supplementary file 16 — Reporting Summary [file 41467_2023_42812_MOESM16_ESM.pdf]

nature portfolio

Corresponding author(s): Jinqing Huang

Last updated by author(s): Sep 26, 2023

Reporting Summary

Nature Portfolio wishes to improve the reproducibility of the work that we publish. This form provides structure for consistency and transparency in reporting. For further information on Nature Portfolio policies, see our Editorial Policies and the Editorial Policy Checklist.

Statistics

For all statistical analyses, confirm that the following items are present in the figure legend, table legend, main text, or Methods section.

n/a	Confirmed	
☐	☒	The exact sample size (*n*) for each experimental group/condition, given as a discrete number and unit of measurement
☐	☒	A statement on whether measurements were taken from distinct samples or whether the same sample was measured repeatedly
☐	☒	The statistical test(s) used AND whether they are one- or two-sided<br>*Only common tests should be described solely by name; describe more complex techniques in the Methods section.*
☒	☐	A description of all covariates tested
☒	☐	A description of any assumptions or corrections, such as tests of normality and adjustment for multiple comparisons
☐	☒	A full description of the statistical parameters including central tendency (e.g. means) or other basic estimates (e.g. regression coefficient) AND variation (e.g. standard deviation) or associated estimates of uncertainty (e.g. confidence intervals)
☒	☐	For null hypothesis testing, the test statistic (e.g. *F*, *t*, *r*) with confidence intervals, effect sizes, degrees of freedom and *P* value noted<br>*Give P values as exact values whenever suitable.*
☒	☐	For Bayesian analysis, information on the choice of priors and Markov chain Monte Carlo settings
☒	☐	For hierarchical and complex designs, identification of the appropriate level for tests and full reporting of outcomes
☒	☐	Estimates of effect sizes (e.g. Cohen's *d*, Pearson's *r*), indicating how they were calculated

Our web collection on statistics for biologists contains articles on many of the points above.

Software and code

Policy information about availability of computer code

Data collection	Spectral data was collected using Winspec for the spectrometer (Teledyne Princeton Instruments, USA).<br>Microscope images were collected using Bluelake for the optical tweezers microscope (Lumicks B. V., Netherlands).
Data analysis	Data was analyzed and plotted using OriginPro. The three-dimensional finite-difference time-domain (3D-FD TD) simulation was conducted using FDTD SOLUTIONS provided by Lumerical Solutions, Inc. Molecular Dynamic (MD) simulations were performed using the GROMACS 2022 software package and AMBER 93 force field.

For manuscripts utilizing custom algorithms or software that are central to the research but not yet described in published literature, software must be made available to editors and reviewers. We strongly encourage code deposition in a community repository (e.g. GitHub). See the Nature Portfolio guidelines for submitting code & software for further information.

Data

Policy information about availability of data

All manuscripts must include a data availability statement. This statement should provide the following information, where applicable:

- Accession codes, unique identifiers, or web links for publicly available datasets
- A description of any restrictions on data availability
- For clinical datasets or third party data, please ensure that the statement adheres to our policy

All data generated in this study are presented in the main text and the supplementary information. Source Data file has been deposited in Figshare under accession

code: https://doi.org/10.6084/m9.figshare.23591901.

Research involving human participants, their data, or biological material

Policy information about studies with human participants or human data. See also policy information about sex, gender (identity/presentation), and sexual orientation and race, ethnicity and racism.

Reporting on sex and gender	*Use the terms sex (biological attribute) and gender (shaped by social and cultural circumstances) carefully in order to avoid confusing both terms. Indicate if findings apply to only one sex or gender; describe whether sex and gender were considered in study design; whether sex and/or gender was determined based on self-reporting or assigned and methods used. Provide in the source data disaggregated sex and gender data, where this information has been collected, and if consent has been obtained for sharing of individual-level data; provide overall numbers in this Reporting Summary. Please state if this information has not been collected. Report sex- and gender-based analyses where performed, justify reasons for lack of sex- and gender-based analysis.*
Reporting on race, ethnicity, or other socially relevant groupings	*Please specify the socially constructed or socially relevant categorization variable(s) used in your manuscript and explain why they were used. Please note that such variables should not be used as proxies for other socially constructed/relevant variables (for example, race or ethnicity should not be used as a proxy for socioeconomic status). Provide clear definitions of the relevant terms used, how they were provided (by the participants/respondents, the researchers, or third parties), and the method(s) used to classify people into the different categories (e.g. self-report, census or administrative data, social media data, etc.) Please provide details about how you controlled for confounding variables in your analyses.*
Population characteristics	*Describe the covariate-relevant population characteristics of the human research participants (e.g. age, genotypic information, past and current diagnosis and treatment categories). If you filled out the behavioural & social sciences study design questions and have nothing to add here, write "See above."*
Recruitment	*Describe how participants were recruited. Outline any potential self-selection bias or other biases that may be present and how these are likely to impact results.*
Ethics oversight	*Identify the organization(s) that approved the study protocol.*

Note that full information on the approval of the study protocol must also be provided in the manuscript.

Field-specific reporting

Please select the one below that is the best fit for your research. If you are not sure, read the appropriate sections before making your selection.

☒ Life sciences ☐ Behavioural & social sciences ☐ Ecological, evolutionary & environmental sciences

For a reference copy of the document with all sections, see nature.com/documents/nr-reporting-summary-flat.pdf

Life sciences study design

All studies must disclose on these points even when the disclosure is negative.

Sample size	hIAPP containing the disulfide bridge between Cys-2 and Cys-7 and the amidated C-terminal was purchased from GL Biochem (Shanghai) Ltd. It was dissolved in hexafluoro-isopropanol (HFIP) at a concentration of 1 mg/mL and incubated for 1 h, followed by lyophilization. Then, the purified hIAPP was rehydrated in 10 mM PBS buffer and filtered through a 0.22 nm Tuffryn syringe filter to prepare the stock solutions with a concentration of 1 mg/mL (250 µM). In parallel experimental sessions, several batches of the hIAPP stock solutions were prepared as independent samples for the subsequent incubations at different pH conditions. Parts of the hIAPP stock solutions were diluted to the concentration of 10 µM and adjusted to pH 5.5, followed by the 24-hour incubation at 37 °C. Parts of the hIAPP stock solutions were diluted to the concentration of 10 µM and adjusted to pH 7.4, followed by the 24-hour incubation at 37 °C. Parts of the hIAPP stock solutions were diluted to the concentration of 10 µM and adjusted to pH 7.4 for a 2-hour incubation at 37 °C, followed by the subsequent adjustment from pH 7.4 to 5.5 to continue the succeeding 22-hour incubation at 37 °C. Meanwhile, the time-dependent CD, SERS, and AFM characterizations were conducted repeatedly for parallel experimental sessions during the incubations of these hIAPP solutions from different batches of independent preparations.
Data exclusions	No data was excluded. All the raw data were provided.
Replication	To characterize the structures of hIAPP, 10000 SERS spectra were collected at each early timepoint during the incubations under different pH conditions in multiple parallel experimental sessions for statistical analysis. For the BiASERS analysis of MB and NBA at dynamic nanocavity. 3600 SERS measurements were conducted across twenty parallel sessions. Representative spectra were observed more than 10 times in parallel SERS measurements. Micrographs are representative of at least three independent experiments.
Randomization	Randomization was not applicable since there were no experimental groups in the study.
Blinding	Blinding was not applicable since there were no experimental groups in the study.

Reporting for specific materials, systems and methods

We require information from authors about some types of materials, experimental systems and methods used in many studies. Here, indicate whether each material, system or method listed is relevant to your study. If you are not sure if a list item applies to your research, read the appropriate section before selecting a response.

Materials & experimental systems

n/a	Involved in the study
☒	☐ Antibodies
☒	☐ Eukaryotic cell lines
☒	☐ Palaeontology and archaeology
☒	☐ Animals and other organisms
☒	☐ Clinical data
☒	☐ Dual use research of concern
☒	☐ Plants

Methods

n/a	Involved in the study
☒	☐ ChIP-seq
☒	☐ Flow cytometry
☒	☐ MRI-based neuroimaging
